# Association of Dietary Resistant Starch Intake with Obesity and Metabolic Syndrome in Korean Adults

**DOI:** 10.3390/nu16010158

**Published:** 2024-01-03

**Authors:** Min-Sook Kang, Kyeong-A Jang, Haeng-Ran Kim, SuJin Song

**Affiliations:** 1 Department of Agro-Food Resources, National Institute of Agricultural Sciences, Rural Development Administration, 166 Nongsaengmyeong-ro, Wanju-gun 55365, Republic of Korea; mskang0803@korea.kr (M.-S.K.); jka1213@korea.kr (K.-A.J.); kimhrr@korea.kr (H.-R.K.); 2 Department of Food and Nutrition, Hannam University, 1646 Yuseong-daero, Yuseong-gu, Daejeon 34054, Republic of Korea

**Keywords:** resistant starch, diet, metabolic syndrome, obesity, Korea

## Abstract

Research findings on the relationship between dietary resistant starch (RS) intake and metabolic diseases using population-based data are very scarce. This study examined the association of dietary RS intake with obesity and metabolic syndrome in Korean adults. A total of 12,491 adults (5292 men and 7199 women) were selected from the 2016–2018 Korea National Health and Nutrition Examination Survey data. The individual RS intake (g) was calculated by linking the 1-day 24 h recall data with the RS content database for common Korean foods. Obesity was defined as a BMI ≥ 25.0 kg/m^2^. Metabolic syndrome was defined as having three or more of the following: abdominal obesity, elevated triglycerides, low HDL cholesterol, elevated fasting blood glucose, and elevated blood pressure. Odds ratios (ORs) with 95% confidence intervals (CIs) for obesity and metabolic syndrome across quartiles (Qs) of RS intake were calculated using multiple logistic regression analysis. In men, the highest quartile of RS intake showed a significantly lower OR for metabolic syndrome compared to the lowest quartile after adjusting for covariates (OR = 0.71, 95% CI = 0.56–0.92, *p*-trend = 0.0057). Dietary RS intake in men was also inversely associated with obesity (Q4 vs. Q1: OR = 0.80, 95% CI = 0.67–0.97, *p*-trend = 0.0329) and elevated triglycerides (Q4 vs. Q1: OR = 0.80, 95% CI = 0.66–0.98, *p*-trend = 0.0314). In women, RS intake was not associated with metabolic syndrome. Our findings may serve as useful data for developing guidelines for RS intake and conducting further cohort and clinical studies to investigate the health effects of RS.

## 1. Introduction

Resistant starch (RS) is defined as starch that cannot be digested or absorbed in the small intestines of healthy individuals and includes the sum of its degradation products [1]. RS is also referred to as indigestible starch and thus exhibits physiological effects similar to those of dietary fibers [2]. RS is classified into five types: RS1 is starch physically trapped in a cellular structure, preventing enzymatic degradation; RS2 is starch within a crystal structure that resists enzymatic activity and is characterized by the loss of resistance upon gelatinization; RS3 is retrograded starch; its amylose undergoes crystallization to acquire resistance to enzymes, such as when heat-gelatinized starch cools; RS4 is a chemically denatured indigestible starch; and RS5 is the amylose–lipid complex generated from starch processing [3].

Following the various health benefits of RS in humans, research interest in it has been increasing. Recent studies have investigated the protective effects of RS supplementation against metabolic disorders in clinical settings [4,5,6,7,8,9,10,11]. RS produces less energy, approximately 2.7 kcal/g [9], and reduces appetite [10]; thus, it might help with weight management. Additionally, previous meta-analyses have revealed that RS contributes to the regulation of the blood glucose and insulin concentrations [4,5,6,7], improvement in the blood lipid levels [8], and reductions in inflammatory markers [11]. Thus, a diet rich in RS could improve metabolic diseases, such as obesity, type 2 diabetes, dyslipidemia, and metabolic syndrome.

Although previous clinical studies have verified the beneficial effects of RS supplementation on health outcomes, few nutritional epidemiologic studies have investigated a level of dietary RS intake in a given population and its associations with metabolic diseases. This is primarily due to the absence of a database for the RS contents in foods commonly consumed by a target population. Several previous studies have attempted to estimate the actual dietary RS intake, and they report a range of from 3.2 g/day to 14.9 g/day in different countries [12,13,14,15,16,17]. However, findings on the relationship between dietary RS intake and metabolic diseases observed from population-based data are very scarce.

Most recently, a database of RS contents for common Korean foods has been developed [18]. Using the newly developed database of RS contents, the mean intake of dietary RS was estimated at approximately 3.1 g/day for Korean adults (3.3 g in men and 2.8 g in women), and the RS intake by Korean adults was shown to be lower compared to that in other countries [18]. Previous studies in Korean adults showed that a high-carbohydrate diet or a low intake of dietary fiber was positively associated with metabolic syndrome and its components [19,20]. Additionally, prior research has demonstrated differences in dietary practices, lifestyles, and metabolic diseases between men and women and shown sex-related variations in the associations of dietary carbohydrates with health outcomes [19,20]. Dietary RS intake as a fraction of dietary carbohydrates might be predicted to be significantly related to obesity and metabolic syndrome in a given population, and differentially associated with health outcomes by sex.

In Korea, the prevalence of metabolic syndrome is increasing, and about one-third of Korean adults aged 30 years and older had metabolic syndrome between 2016 and 2018 [21]. Furthermore, in 2021, heart and cerebrovascular diseases were ranked as the second and fourth causes of death, respectively [22]. Identifying protective dietary factors and suggesting effective intake strategies are necessary to prevent and manage metabolic diseases. Therefore, this study examined the associations of dietary RS intake with obesity and metabolic syndrome in Korean men and women using data from the 2016–2018 Korea National Health and Nutrition Examination Survey (KNHANES) to expand the research scope of RS and investigate the role of dietary RS intake on health outcomes.

## 2. Materials and Methods

### 2.1. Data and Study Subjects

This cross-sectional study used data from the 2016–2018 KNHANES. The KNHANES is an ongoing surveillance system that assesses the nutritional health status and prevalence of chronic diseases in a large and nationally representative sample of Koreans. The KNHANES is conducted by the Korea Disease Control and Prevention Agency every year and consists mainly of a health interview, health examination, and nutrition survey. Detailed information on the KNHANES is available elsewhere [23].

Among the 24,269 participants in the 2016–2018 KNHANES, 17,338 adults aged 19–74 years were eligible for this study. Of the eligible study participants, (1) individuals who had no dietary data (*n* = 2362), (2) individuals with missing data on the anthropometric or biochemical variables (*n* = 1550), (3) individuals with missing data on the sociodemographic or lifestyle variables (*n* = 543), (4) pregnant or lactating women (*n* = 162), and (5) individuals whose energy intakes were <500 kcal or >5000 kcal [24,25] (*n* = 230) were excluded. Data from 12,491 participants (5292 men and 7199 women) were included in the final analysis. This study was performed in line with the principles of the Declaration of Helsinki and was approved by the Institutional Review Board of the Korea Disease Control and Prevention Agency (No. 2018-01-03-P-A, 12 January 2018). Written informed consent was obtained from all the study subjects.

### 2.2. Assessment of Dietary RS Intake

In this study, dietary data from a single 24 h dietary recall were used. The 24 h dietary recall was conducted as part of the nutrition survey of the KNHANES by trained dietitians in the participants’ households. Participants were asked to provide detailed information on all foods and beverages consumed in the past 24 h through a standardized and structured interview. During the interviews, trained dietitians used food models, pictures, and other visual aids to help the participants recall the types and amounts of foods and beverages they consumed. The dietary data obtained were linked to a food composition table (9.1 revision) established by the National Institute of Agricultural Sciences of Korea [26] to calculate the energy and nutrient intakes (e.g., carbohydrates, protein, fat, vitamin A, thiamin, riboflavin, niacin, vitamin C, calcium, phosphorus, sodium, potassium, and iron) for each subject. Additionally, the dietary fiber intake for each subject was assessed based on a dietary fiber composition table developed to evaluate the dietary fiber intake of Koreans who participated in the KNHANES [27]. The macronutrient intake was calculated as the proportion of energy intake obtained from each macronutrient, and the intake of vitamins and minerals was calculated as the intake density per 1000 kcal.

A newly developed database of RS contents in common Korean foods was used to assess the dietary RS intake in this study population [18]. The RS database contains the RS content per 100 g of each food for 3885 food items. The RS database was established based on analytical, reference, calculated, imputed, and food recipe values collected from the direct analyses of food samples, as well as from previous studies conducted in Korea and other countries. Detailed information on the RS database is described elsewhere [18]. Briefly, analytical values of the RS contents were obtained from a direct analysis of food samples and previous studies conducted in Korea through a literature review. Reference values for the RS contents were collected from previous studies that analyzed overseas samples through a literature review. For foods with analytical or reference values for RS contents that could not be used, calculated or imputed values were applied. When several foods constituted a single food item (e.g., sandwich, hamburger, dumpling, and pizza), the contents of the main food ingredients within the food were calculated based on food recipes. The RS content value corresponding to the weight of the main food ingredient was calculated and applied as a food recipe value. Based on previous studies, the RS content was assumed to be 0 for meat, poultry, fish, shellfish, eggs, milk, dairy products, fats and oils, alcohol, sugars and sweets, sugar-sweetened beverages, coffee and teas, sauces and seasonings, and certain fruits and vegetables, as these food items have very low carbohydrate contents or contain carbohydrates as simple sugars [15,27,28]. The 24 h recall data from the 2016–2018 KNHANES and the RS content databases were linked based on the KNHANES primary food codes. After calculating the RS intake for each food item on the list consumed by each participant, the individual RS intake per day was calculated (g). The dietary RS intake was grouped into quartiles according to sex for further analysis.

### 2.3. Definition of Metabolic Syndrome and Its Components

Information on the anthropometric and biochemical variables was obtained through the health examination of the KNHANES. Height (SECA 274, SECA, Hamburg, Germany) and weight (InBody 970, InBody, Seoul, Korea) were measured using standardized techniques and calibrated equipment. Body mass index (BMI) was calculated from the measured height and weight (kg/m^2^). Obesity was defined as a BMI ≥ 25 kg/m^2^ [29]. Waist circumference was measured to the nearest 0.1 cm using a measuring tape in a standing position at the umbilical level (SECA 200, SECA, Hamburg, Germany). Blood pressure was measured three times using standard methods (WatchBP Office AFIB, Microlife, Widnau, Switzerland), and the average of the last two values was used. Venous blood samples after fasting for at least 8 h were collected from each subject by trained medical personnel and were analyzed in a certified clinical laboratory. High-density lipoprotein (HDL) cholesterol, triglycerides, and fasting blood glucose levels were measured via enzymatic methods using a Hitachi automatic analyzer 7600-210 (Hitachi, Tokyo, Japan).

Metabolic syndrome was defined, according to the clinical practice guideline published by the Korean Society of Cardiometabolic Syndrome, if any three or more of the following components were present [30]: abdominal obesity (waist circumference ≥ 90 cm in men and ≥85 cm in women); elevated triglycerides (≥150 mg/dL); low HDL cholesterol (<40 mg/dL in men and <50 mg/dL in women); elevated blood glucose (fasting blood glucose ≥ 100 mg/dL or taking medication or using insulin therapy); and elevated blood pressure (systolic blood pressure ≥ 130 mmHg or diastolic blood pressure ≥ 85 mmHg, or taking medication).

### 2.4. Measurement of Sociodemographic and Lifestyle Variables

Sociodemographic (e.g., living area, household income, and education) and lifestyle (e.g., smoking, alcohol consumption, and physical activity) information was collected using a structured questionnaire in the health interview of the KNHANES. Living areas were classified as urban and rural areas. Household income was grouped into the lowest, medium–low, medium–high, and highest groups. Education was categorized as elementary school, middle school, high school, and college or higher. Current smoking was defined as “yes” if a participant had smoked 100 cigarettes during their lifetime and was smoking regularly or occasionally at the time of the survey. Current alcohol consumption was defined as “yes” if the subject drank one glass of alcohol or more per month in the previous year. Physical activity was determined as “yes” if a subject engaged in high-intensity exercise for ≥75 min or moderate physical activity for ≥150 min in the previous week.

### 2.5. Statistical Analyses

Statistical analyses were conducted using Statistical Analysis Systems (SAS) software, version 9.4 (SAS Institute, Cary, NC, USA). To produce estimates of the entire Korean adult population from the representative survey sample of the KNHANES, all analyses performed in this study accounted for the effects of the complex sampling design and used appropriate sampling weights using the PROC SURVEY in SAS. All analyses in this study were conducted by sex. The characteristics of the study subjects are presented as frequencies and percentages for categorical variables (e.g., sociodemographic and lifestyle variables and the prevalence of metabolic syndrome and its components) and as means with standard errors (SE) for continuous variables (e.g., BMI, energy and nutrient intakes). To determine the differences in these variables by sex, the chi-square test was used for categorical variables, and the *t*-test was used for continuous variables.

The sociodemographic and lifestyle variables across the quartiles of dietary RS intake are presented as frequencies and percentages, and the differences in these variables were tested using the chi-square test. The energy and nutrient intakes across quartiles of dietary RS intake are presented as means and SEs, and the differences in these variables were tested using a multiple regression model. Multiple logistic regression analysis was performed to estimate the odds ratios (ORs) and 95% confidence intervals (CIs) for metabolic syndrome and its components by the quartiles of dietary RS intake, with the lowest-quartile group as the reference group. Age (continuous), living area (urban or rural), household income (lowest, medium–low, medium–high, or highest), education (elementary, middle school, high school, college or higher), current smoking status (yes or no), current alcohol consumption (yes or no), physical activity (yes or no), total energy intake (continuous), and BMI (continuous) were considered potential confounding variables and were controlled for in all models. To calculate the *p*-trends for metabolic syndrome and its components according to the quartiles of dietary RS intake, the median value of the RS in each quartile was entered as a continuous variable in the logistic regression model. Statistical significance was set at *p* < 0.05.

## 3. Results

### 3.1. Characteristics of Study Subjects by Sex

Table 1 shows the characteristics of the study population according to sex. Of the study subjects, about 49% were men, and the mean age was 44.7 years (SE = 0.3) for men and 45.8 years (SE = 0.2) for women. Men showed higher proportions of individuals who had higher household income and education levels, currently smoked and drank alcohol, and engaged in physical activity than those in women (*p*-values < 0.05). The prevalence of metabolic syndrome was 24.9% in the study population and 30.7% and 19.4% in men and women, respectively (*p*-value < 0.0001). The prevalence of the obesity and metabolic syndrome components was higher in men than women, except for low HDL cholesterol (*p*-values < 0.0001). Men had significantly higher intakes of energy, macronutrients, and dietary fiber than women. The mean RS intake was estimated at 3.14 g (SE = 0.06) for adults in this study. The dietary RS intake was higher in men than women (3.40 g in men and 2.87 g in women; *p*-value < 0.0001).

### 3.2. Sociodemographic and Lifestyle Characteristics across Quartiles of Dietary RS Intake by Sex

Table 2 shows the sociodemographic and lifestyle variables across the quartiles of dietary RS intake by sex. Among men, the proportion of individuals with ≥college education was higher in the highest quartile of dietary RS intake (Q4 vs. Q1: 51% vs. 45.6%, *p*-value = 0.0007). In women, the proportions of individuals who were older and had higher household income and education levels were higher in the highest quartile of dietary RS intake than those in the lowest quartile (*p*-values < 0.05). The other sociodemographic and lifestyle variables were not associated with dietary RS intake in men or women.

### 3.3. Energy and Nutrient Intakes across Quartiles of Dietary RS Intake by Sex

The energy and nutrient intakes across the quartiles of dietary RS intake according to sex are presented in Table 3. In both men and women, individuals in the highest-quartile group of dietary RS intake had higher intakes of total energy, carbohydrates (% of energy), and dietary fiber (g/1000 kcal), with lower intakes of protein (% of energy), fat (% of energy), vitamin A (μg RAE/1000 kcal), thiamin (mg/1000 kcal), niacin (mg/1000 kcal), phosphorus (mg/1000 kcal), and iron (mg/1000 kcal), compared to those in the lowest-quartile group. The intakes of riboflavin and sodium (mg/1000 kcal) significantly increased across the quartiles of dietary RS intake in men but not in women. Calcium intake (mg/1000 kcal) was inversely associated with dietary RS intake in women. In all study participants, vitamin C and potassium intakes (mg/1000 kcal) were not associated with dietary RS intake.

### 3.4. Association between Dietary RS Intake and Metabolic Syndrome by Sex

Table 4 shows the ORs (95% CIs) for metabolic syndrome across the quartiles of dietary RS intake by sex. Among Korean men, the highest quartile of dietary RS intake showed an OR of 0.71 for metabolic syndrome compared to the lowest quartile in model 1 (95% CI = 0.56–0.92, *p*-trend = 0.0057). After additional adjustment for dietary fiber intake in model 2, a decreasing trend in the OR for metabolic syndrome across the quartiles of dietary RS intake was still significant (*p*-trend = 0.0483). In Korean women, dietary RS intake was not significantly associated with metabolic syndrome.

### 3.5. Association of Dietary RS Intake with Obesity and Metabolic Syndrome Components by Sex

Table 5 presents the ORs (95% CIs) for the obesity and metabolic syndrome components across the quartiles of dietary RS intake by sex. In men, the dietary RS intake was inversely associated with obesity (Q4 vs. Q1: OR = 0.80, 95% CI = 0.67–0.97, *p*-trend = 0.0329) and elevated triglycerides (Q4 vs. Q1: OR = 0.80, 95% CI = 0.66–0.98, *p*-trend = 0.0314) after adjusting for potential confounding variables. Dietary RS intake in men was not associated with abdominal obesity, low HDL cholesterol, or elevated fasting blood glucose. In both men and women, a higher intake of dietary RS showed a decreasing trend in the ORs for elevated blood pressure (Q4 vs. Q1: OR = 0.78, 95% CI = 0.64–0.97, *p*-trend = 0.0536 in men; Q4 vs. Q1: OR = 0.81, 95% CI = 0.65–1.00, *p*-trend = 0.0207 in women). Obesity and other metabolic syndrome components did not show any association with dietary RS intake in women.

## 4. Discussion

This study examined the association of dietary RS intake with obesity and metabolic syndrome using a large and nationally representative sample of Korean adults from the 2016–2018 KNHANES. In Korean men, a higher dietary RS intake was inversely associated with metabolic syndrome after adjusting for potential confounding variables. This association was explained by the significant inverse association of dietary RS intake with obesity and elevated triglycerides levels in men. However, in Korean women, dietary RS intake was not associated with metabolic diseases.

This study assessed the dietary RS intake of Korean adults using the newly developed RS database for common Korean foods and showed the mean intake of dietary RS to be 3.40 g in men and 2.87 g in women. Because Koreans consume cereal grains and their products, such as white rice, noodles, and bread, as staple foods in their daily diets, over half of the RS intake by the study participants came from these food sources [18]. The RS database used in this study was constructed using information collected on the RS contents of foods from analytical, reference, calculated, imputed, or recipe-based values. At present, there is no official database of RS contents. In previous studies, data on RS contents were collected for certain frequently consumed foods in each target population to evaluate the RS intake. In previous studies conducted in the US, RS content values were assigned to foods commonly consumed by US citizens based on a systematic review of studies containing RS content data [15,31]. The estimates of the RS intakes reported from different countries ranged from 3.2 g/day to 14.9 g/day [12,13,14,15,16,17,32,33]. As the studies varied in terms of the study periods and designs, subject characteristics, and RS measurement and dietary assessment methods, it was difficult to directly compare the RS intakes between studies. Nevertheless, the RS intake in Korean adults is lower than that in other countries.

In this study, Korean men in the highest RS intake quartile had a 29% lower risk of having metabolic syndrome than those in the lowest quartile. The association was weakened after additional adjustment for dietary fiber intake but was still significant. Significant inverse associations of dietary RS intake with obesity and elevated triglyceride levels were also observed in men. RS produces less energy than digestible starch (2.7 kcal/g vs. 4 kcal/g) [9], and RS consumption may reduce appetite by delaying gastric emptying and improving glycemic control [10]. In addition, recent systematic reviews and meta-analyses have consistently reported that RS supplementation improves blood glucose and insulin levels, as well as blood lipid profiles [4,5,6,7,8]. RS is also known to be fermented in the large intestine by microbiota that produce short-chain fatty acids (SCFAs), and these SCFAs may promote insulin secretion and increase insulin sensitivity by stimulating the production of insulin-related hormones, consequently controlling fasting blood glucose levels [7]. Additionally, SCFAs may increase cholesterol excretion and inhibit hepatic cholesterol synthesis [8]. The improvement in glucose and lipid metabolism following RS consumption can also be explained by the production of beneficial gut bacteria in the large intestine [34]. Through these underlying mechanisms, dietary RS intake may ameliorate metabolic syndrome, which is characterized by the coexistence of cardiovascular risk factors and its components, such as obesity and hypertriglyceridemia.

The reduced risks for obesity and metabolic syndrome in men was likely due to a synergistic effect of several food components in their diets. The key food sources of RS intake in the diets of the study subjects were whole grains, legumes, fruits, nuts, and seeds. Therefore, RS intake in combination with these healthy food components appears to play an important role in Korean men to have an inverse association with metabolic syndrome and obesity. This is a holistic approach showing the effect of RS intake on health outcomes because it is an epidemiological approach, rather than a reductionist approach in a clinical study. However, Korean women showed no association of dietary RS intake with metabolic diseases in this study, and thus additional studies are needed.

Interestingly, our analysis revealed a decreasing tendency in the prevalence of elevated blood pressure with increasing dietary RS intake in both men and women. The explanation for this association remains unclear due to the limited research on RS intake and blood pressure. The potential role of RS intake in reducing blood pressure may be explained by the improvement in insulin sensitivity, the vascular endothelial function, and mineral absorption in the intestine [35,36]. Furthermore, as the study population mainly obtained dietary RS from whole grains and legumes, which contain high amounts of dietary fiber, magnesium, and potassium [18], the synergistic effect of RS intake and these protective nutrients on blood pressure can also be considered.

The adequate amount of dietary RS intake to demonstrate improvement in metabolic risk factors has not yet been defined. However, approximately 15–20 g/day is considered appropriate for beneficial health effects, based mainly on clinical studies [14,31,37]. The RS intake in Korean adults can be improved by appropriately selecting foods with high RS contents. For example, functional rice cultivars with high RS contents can be selected for intake [18]. When cooked potatoes, pasta, or legumes are consumed at low temperatures (e.g., cold salads), the formation of RS3 can ensure an increased intake of RS [3]. For processed foods, those with higher RS contents or added RS could be selected to increase RS intake. Moreover, previous studies have emphasized the intake of whole grains, legumes, and bread or pasta produced from barley, rye, and durum wheat to increase the RS content as well as the dietary fiber intake in the diet [14,33]. Further studies should be conducted to determine the adequate amount of dietary RS required to yield beneficial health effects.

Our study had several limitations. First, the data used were based on a cross-sectional design; therefore, it was difficult to investigate the causal relationship between dietary RS intake and metabolic syndrome. Second, as the dietary data analyzed in this study were collected using a single 24 h dietary recall, they do not represent the usual dietary intake of the study subjects. Third, we did not consider the association with metabolic syndrome according to the RS types due to limited information on the RS compositions of the foods in the RS database. Nevertheless, to the best of our knowledge, this study is the first to examine the association of dietary RS intake with metabolic syndrome and its components using population-based data including a large, nationally representative sample of Koreans. Our findings may contribute to the establishment of guidelines for healthy RS intake and provide basic data for conducting further cohort or clinical studies to investigate the effects of RS intake on the development of metabolic diseases.

## 5. Conclusions

In conclusion, this study found that a higher dietary RS intake was associated with a reduced risk of metabolic syndrome in Korean men. The inverse association between dietary RS intake and metabolic syndrome among men was supported by the decreased prevalence of obesity and hypertriglyceridemia across the quartiles of RS intake. However, there was no association between dietary RS intake, obesity, and metabolic syndrome in Korean women. Because Korean adults frequently consume cereal grains and their products and starch-rich foods as staple foods, our findings might help suggest dietary guidelines for healthy RS intake to prevent and manage metabolic syndrome in this population. Additionally, we anticipate an extended scope of research on RS based on our findings, which could provide basic data for epidemiological and clinical studies aimed at identifying the association between RS intake and health status or metabolic diseases. Prospective cohort and clinical studies are needed to examine the mechanisms underlying the association of dietary RS intake with metabolic syndrome and its components in this population.

## Figures and Tables

**Table 1:** Characteristics of study subjects selected from the 2016–2018 Korea National Health and Nutrition Examination Survey data ^1^.

	Total (*n* = 12,491)		Men (*n* = 5292)		Women (*n* = 7199)		
	*n*	%	*n*	%	*n*	%	*p*-Value ^2^
Sex							
Men	5292	48.9					
Women	7199	51.1					
Age group							
19–49 years	6295	59.5	2638	60.3	3657	58.7	0.0750
50–74 years	6196	40.5	2654	39.7	3542	41.3	
Living area							
Urban	10,352	86.2	4325	85.6	6027	86.8	0.0510
Rural	2139	13.8	967	14.4	1172	13.2	
Household income							
Lowest	1747	12.2	689	11.5	1058	12.9	0.0016
Medium–low	3103	23.8	1275	22.8	1828	24.8	
Medium–high	3654	30.7	1575	31.2	2079	30.1	
Highest	3987	33.3	1753	34.5	2234	32.2	
Education							
Elementary school or less	1924	10.9	616	8.0	1308	13.6	<0.0001
Middle school	1269	8.5	533	7.9	736	9.1	
High school	4234	36.4	1833	36.8	2401	36.0	
College or higher	5064	44.2	2310	47.3	2754	41.3	
Current smoking							
Yes	2161	20.2	1800	35.5	361	5.7	<0.0001
No	10,330	79.8	3492	64.5	6838	94.3	
Current alcohol consumption							
Yes	7037	60.4	3814	72.8	3223	48.6	<0.0001
No	5454	39.6	1478	27.2	3976	51.4	
Physical activity							
Yes	5716	48.6	2552	51.3	3164	46.0	<0.0001
No	6775	51.4	2740	48.7	4035	54.0	
Metabolic syndrome							
Yes	3421	24.9	1764	30.7	1657	19.4	<0.0001
No	9070	75.1	3528	69.3	5542	80.6	
Obesity							
Yes	4295	34.5	2231	42.6	2064	26.8	<0.0001
No	8196	65.5	3061	57.4	5135	73.2	
Abdominal obesity							
Yes	3528	27.0	1770	32.7	1758	21.5	<0.0001
No	8963	73.0	3522	67.3	5441	78.5	
Elevated triglycerides							
Yes	3558	28.7	2092	39.4	1466	18.5	<0.0001
No	8933	71.3	3200	60.6	5733	81.5	
Low HDL cholesterol							
Yes	4038	29.9	1385	25.1	2653	34.5	<0.0001
No	8453	70.1	3907	74.9	4546	65.5	
Elevated fasting blood glucose							
Yes	4355	31.9	2284	38.5	2071	25.5	<0.0001
No	8136	68.1	3008	61.5	5128	74.5	
Elevated blood pressure							
Yes	4778	34.8	2470	43.1	2308	26.9	<0.0001
No	7713	65.2	2822	56.9	4891	73.1	
	Mean	SE	Mean	SE	Mean	SE	*p*-value ^3^
Body mass index (kg/m^2^)	23.9	0.04	24.7	0.06	23.2	0.06	<0.0001
Energy and nutrient intakes							
Energy (kcal)	2032	10.9	2355	15.2	1706	9.9	<0.0001
Carbohydrates (g)	300.3	1.6	335.1	2.2	265.1	1.6	<0.0001
Protein (g)	73.7	0.5	85.6	0.7	61.5	0.5	<0.0001
Fat (g)	46.6	0.4	53.6	0.6	39.5	0.4	<0.0001
Dietary fiber (g)	25.4	0.2	27.0	0.3	23.7	0.2	<0.0001
Resistant starch (g)	3.14	0.06	3.40	0.07	2.87	0.08	<0.0001

^1^ All statistical analyses accounted for the complex sampling design effect and appropriate sampling weights of the national survey using PROC SURVEY in the SAS software. ^2^ *p*-values were obtained from the chi-square test. ^3^ *p*-values were obtained from the *t*-test.

**Table 2:** Sociodemographic and lifestyle characteristics across quartiles of dietary resistant starch (RS) intake in Korean men and women from the 2016–2018 Korea National Health and Nutrition Examination Survey data ^1^.

	Quartiles (Qs) of Dietary RS Intake								
	Q1		Q2		Q3		Q4		*p*-Value ^2^
	*n*	%	*n*	%	*n*	%	*n*	%	
Men	(*n* = 1323)		(*n* = 1323)		(*n* = 1323)		(*n* = 1323)		
RS intake (g)									
Mean ± SE	1.05 ± 0.01		2.00 ± 0.01		3.23 ± 0.01		7.25 ± 0.20		
Interquartile range	0.83–1.32		1.76–2.22		2.82–3.61		4.71–7.26		
Age group									
19–49 years	666	61.6	620	59.7	663	62.7	689	64.7	0.0981
50–74 years	657	38.4	703	40.3	660	37.3	634	35.3	
Living area									
Urban	1094	88.0	1077	87.4	1077	86.0	1077	87.2	0.5675
Rural	229	12.0	246	12.6	246	14.0	246	12.8	
Household income									
Lowest	196	11.7	163	9.9	179	11.6	151	10.6	0.1424
Medium–low	333	23.1	313	21.3	333	24.4	296	20.1	
Medium–high	360	29.3	412	33.8	378	29.1	425	32.2	
Highest	434	35.9	435	35.0	433	34.9	451	37.2	
Education									
Elementary school or less	169	8.6	173	8.6	157	7.4	117	5.2	0.0007
Middle school	139	7.5	151	8.5	129	7.7	114	5.5	
High school	475	38.4	425	34.2	466	38.3	467	38.2	
College or higher	540	45.6	574	48.7	571	46.5	625	51.0	
Current smoking									
Yes	508	39.0	434	34.5	439	35.0	419	34.1	0.0759
No	815	61.0	889	65.5	884	65.0	904	65.9	
Current alcohol consumption									
Yes	981	75.6	951	72.2	934	72.3	948	72.3	0.2108
No	342	24.4	372	27.8	389	27.7	375	27.7	
Physical activity									
Yes	621	51.0	623	50.1	649	52.8	659	54.7	0.1767
No	702	49.0	700	49.9	674	47.2	664	45.3	
Women	(*n* = 1799)		(*n* = 1800)		(*n* = 1800)		(*n* = 1800)		
RS intake (g)									
Mean ± SE	0.83 ± 0.01		1.64 ± 0.01		2.68 ± 0.01		6.52 ± 0.31		
Interquartile range	0.65–1.05		1.43–1.83		2.35–2.99		3.94–6.15		
Age group									
19–49 years	962	60.3	944	59.3	892	56.7	859	54.4	0.0040
50–74 years	837	39.7	856	40.7	908	43.3	941	45.6	
Living area									
Urban	1514	88.7	1490	87.3	1496	87.7	1527	89.3	0.3236
Rural	285	11.3	310	12.7	304	12.3	273	10.7	
Household income									
Lowest	291	13.4	273	13.5	287	13.3	207	10.6	0.0134
Medium–low	475	26.1	480	25.5	440	23.6	433	22.0	
Medium–high	502	30.3	507	29.4	510	29.1	560	31.7	
Highest	531	30.2	540	31.6	563	34.1	600	35.7	
Education									
Elementary school or less	355	14.9	345	13.9	327	13.6	281	12.0	0.0202
Middle school	194	10.0	175	8.6	195	9.4	172	7.9	
High school	629	37.5	590	36.2	580	34.7	602	35.5	
College or higher	621	37.6	690	41.3	698	42.3	745	44.6	
Current smoking									
Yes	107	6.4	101	6.7	84	5.3	69	4.7	0.1617
No	1692	93.6	1699	93.3	1716	94.7	1731	95.3	
Current alcohol consumption									
Yes	816	49.7	841	50.0	813	48.5	753	46.3	0.2228
No	983	50.3	959	50.0	987	51.5	1047	53.7	
Physical activity									
Yes	789	47.4	769	44.8	772	45.7	834	49.5	0.0744
No	1010	52.6	1031	55.2	1028	54.3	966	50.5	

^1^ All statistical analyses accounted for the complex sampling design effect and appropriate sampling weights of the national survey using PROC SURVEY in the SAS software. ^2^ *p*-values were obtained from the chi-square test.

**Table 3:** Energy and nutrient intakes across quartiles of dietary resistant starch (RS) intake in Korean men and women from the 2016–2018 Korea National Health and Nutrition Examination Survey data ^1^.

	Quartiles (Qs) of Dietary RS Intake								
	Q1		Q2		Q3		Q4		*p*-Trend ^2^
	Mean	SE	Mean	SE	Mean	SE	Mean	SE	
Men	(*n* = 1323)		(*n* = 1323)		(*n* = 1323)		(*n* = 1323)		
Energy (kcal)	1924	27.3	2224	24.9	2415	24.7	2846	28.2	<0.0001
Carbohydrates (% of energy)	59.9	0.4	63.0	0.4	63.7	0.3	64.5	0.3	<0.0001
Protein (% of energy)	17.5	0.2	15.9	0.1	15.3	0.2	14.7	0.1	<0.0001
Fat (% of energy)	22.6	0.4	21.0	0.3	21.0	0.3	20.8	0.3	<0.0001
Dietary fiber (g/1000 kcal)	11.0	0.2	11.9	0.2	12.1	0.2	12.6	0.2	<0.0001
Vitamin A (μg RAE/1000 kcal)	188.9	7.2	193.6	6.3	176.2	4.9	163.9	3.8	0.0001
Thiamin (mg/1000 kcal)	0.73	0.01	0.71	0.01	0.68	0.01	0.63	0.01	<0.0001
Riboflavin (mg/1000 kcal)	0.77	0.01	0.77	0.01	0.81	0.01	0.81	0.01	0.0008
Niacin (mg/1000 kcal)	7.6	0.1	7.0	0.1	6.7	0.2	6.4	0.1	<0.0001
Vitamin C (mg/1000 kcal)	30.5	1.9	28.8	1.0	28.9	1.0	29.4	1.0	0.6118
Calcium (mg/1000 kcal)	248.7	4.4	259.6	4.5	259.5	3.8	252.0	3.6	0.6656
Phosphorus (mg/1000 kcal)	559.4	5.6	544.7	4.3	525.4	4.9	512.3	4.0	<0.0001
Sodium (mg/1000 kcal)	1704	25.6	1784	23.3	1842	25.3	1770	24.9	0.0226
Potassium (mg/1000 kcal)	1404	19.0	1374	13.4	1365	15.1	1391	14.3	0.3886
Iron (mg/1000 kcal)	6.1	0.1	6.3	0.1	5.9	0.1	5.6	0.1	<0.0001
Women	(*n* = 1799)		(*n* = 1800)		(*n* = 1800)		(*n* = 1800)		
Energy (kcal)	1312	14.8	1593	14.6	1815	16.5	2132	21.2	<0.0001
Carbohydrates (% of energy)	61.5	0.4	64.3	0.3	64.9	0.3	67.1	0.3	<0.0001
Protein (% of energy)	16.1	0.2	15.0	0.1	14.4	0.1	13.8	0.1	<0.0001
Fat (% of energy)	22.3	0.3	20.8	0.3	20.7	0.3	19.1	0.2	<0.0001
Dietary fiber (g/1000 kcal)	13.2	0.2	13.8	0.2	14.3	0.2	15.4	0.2	<0.0001
Vitamin A (μg RAE/1000 kcal)	229.8	6.1	211.3	4.5	215.8	8.6	195.3	4.4	<0.0001
Thiamin (mg/1000 kcal)	0.71	0.01	0.71	0.01	0.67	0.01	0.65	0.01	<0.0001
Riboflavin (mg/1000 kcal)	0.84	0.01	0.84	0.01	0.85	0.01	0.84	0.01	0.3905
Niacin (mg/1000 kcal)	7.7	0.1	7.1	0.1	6.8	0.1	6.7	0.1	<0.0001
Vitamin C (mg/1000 kcal)	36.6	1.2	36.7	1.0	35.1	0.9	39.7	1.1	0.7164
Calcium (mg/1000 kcal)	299.1	4.9	284.5	4.3	284.3	3.9	285.5	4.7	0.0103
Phosphorus (mg/1000 kcal)	592.4	4.8	567.3	4.0	551.4	3.8	544.1	4.1	<0.0001
Sodium (mg/1000 kcal)	1713	26.8	1742	22.6	1751	22.2	1701	25.2	0.9950
Potassium (mg/1000 kcal)	1565	16.1	1536	15.1	1522	14.3	1645	15.1	0.1980
Iron (mg/1000 kcal)	6.5	0.1	6.6	0.1	6.3	0.1	6.3	0.1	<0.0001

^1^ All statistical analyses accounted for the complex sampling design effect and appropriate sampling weights of the national survey using PROC SURVEY in the SAS software. ^2^ *p*-trends were obtained from the multiple regression model after adjusting for age, living area, household income, education, current smoking, current alcohol consumption, physical activity, and body mass index.

**Table 4:** Association between dietary resistant starch (RS) intake and metabolic syndrome in Korean men and women from the 2016–2018 Korea National Health and Nutrition Examination Survey data ^1,2^.

	Quartiles (Qs) of Dietary RS Intake							
	Q1	Q2		Q3		Q4		*p*-Trend
	OR	OR	(95% CI)	OR	(95% CI)	OR	(95% CI)	
Men	(*n* = 1323)	(*n* = 1323)		(*n* = 1323)		(*n* = 1323)		
Model 1 ^3^	1.00	0.94	(0.75–1.19)	0.89	(0.71–1.12)	0.71	(0.56–0.92)	0.0057
Model 2 ^4^	1.00	0.98	(0.78–1.24)	0.94	(0.75–1.19)	0.79	(0.61–1.02)	0.0483
Women	(*n* = 1799)	(*n* = 1800)		(*n* = 1800)		(*n* = 1800)		
Model 1	1.00	0.83	(0.66–1.05)	0.92	(0.74–1.15)	0.90	(0.69–1.17)	0.7357
Model 2	1.00	0.84	(0.66–1.05)	0.94	(0.75–1.17)	0.92	(0.71–1.20)	0.8980

^1^ All statistical analyses accounted for the complex sampling design effect and appropriate sampling weights of the national survey using PROC SURVEY in the SAS software. ^2^ Multiple logistic regression analyses were performed to estimate the odds ratios (ORs), 95% confidence intervals (CIs), and *p*-trends for metabolic syndrome by quartiles of RS intake, with the lowest-quartile group as the reference group. To calculate the *p*-trends across the quartiles of RS intake, the median RS intake in each quartile was entered as a continuous variable in the logistic regression model. ^3^ Model 1 was adjusted for age, living area, household income, education, current smoking, current alcohol consumption, physical activity, total energy intake, and body mass index. ^4^ Model 2 was additionally adjusted for dietary fiber intake from model 1.

**Table 5:** Association of dietary resistant starch (RS) intake with obesity and metabolic syndrome components in Korean men and women from the 2016–2018 Korea National Health and Nutrition Examination Survey data ^1,2^.

	Quartiles (Qs) of Dietary RS Intake							
	Q1	Q2		Q3		Q4		*p*-Trend
	OR	OR	(95% CI)	OR	(95% CI)	OR	(95% CI)	
Men	(*n* = 1323)	(*n* = 1323)		(*n* = 1323)		(*n* = 1323)		
Obesity	1.00	0.90	(0.75–1.07)	1.00	(0.83–1.21)	0.80	(0.67–0.97)	0.0329
Metabolic syndrome components								
Abdominal obesity	1.00	0.89	(0.73–1.09)	1.00	(0.82–1.21)	0.89	(0.73–1.09)	0.4135
Elevated triglycerides	1.00	0.92	(0.76–1.11)	0.82	(0.68–0.99)	0.80	(0.66–0.98)	0.0314
Low HDL cholesterol	1.00	0.91	(0.73–1.13)	0.86	(0.70–1.05)	0.88	(0.70–1.10)	0.3136
Elevated fasting blood glucose	1.00	0.94	(0.76–1.15)	0.91	(0.75–1.11)	0.93	(0.75–1.15)	0.5577
Elevated blood pressure	1.00	0.84	(0.70–1.02)	0.83	(0.69–1.01)	0.78	(0.64–0.97)	0.0536
Women	(*n* = 1799)	(*n* = 1800)		(*n* = 1800)		(*n* = 1800)		
Obesity	1.00	0.82	(0.69–0.98)	0.81	(0.67–0.96)	0.96	(0.80–1.16)	0.8046
Metabolic syndrome components								
Abdominal obesity	1.00	0.94	(0.78–1.13)	0.88	(0.73–1.07)	0.94	(0.76–1.16)	0.6556
Elevated triglycerides	1.00	0.81	(0.66–1.00)	0.93	(0.76–1.13)	0.97	(0.78–1.22)	0.6631
Low HDL cholesterol	1.00	0.94	(0.80–1.12)	0.87	(0.74–1.03)	0.97	(0.80–1.18)	0.9013
Elevated fasting blood glucose	1.00	0.87	(0.72–1.05)	0.96	(0.79–1.18)	1.00	(0.80–1.25)	0.6115
Elevated blood pressure	1.00	1.02	(0.83–1.26)	0.96	(0.78–1.19)	0.81	(0.65–1.00)	0.0207

^1^ All statistical analyses accounted for the complex sampling design effect and appropriate sampling weights of the national survey using PROC SURVEY in the SAS software. ^2^ Multiple logistic regression analyses were performed to estimate the odds ratios (ORs), 95% confidence intervals (CIs), and *p*-trends for the obesity and metabolic syndrome components by quartile of RS intake, with the lowest-quartile group as the reference group. All analyses were adjusted for age, living area, household income, education, current smoking, current alcohol consumption, physical activity, total energy intake, and body mass index (except for obesity and abdominal obesity). To calculate the *p*-trends across the quartiles of RS intake, the median RS intake in each quartile was entered as a continuous variable in the logistic regression model.

## Data Availability

The KNHANES data are available at https://knhanes.kdca.go.kr/knhanes/main.do (accessed on 1 September 2022). These data are free of charge for academic research.
